# Bone Bruise Patterns After Noncontact Anterior Cruciate Ligament Tears Differ Between Alpine Skiers and Pivoting Sports Athletes

**DOI:** 10.1177/03635465251332272

**Published:** 2025-04-22

**Authors:** Steffen T. Ubl, Romed P. Vieider, Jesse Seilern und Aspang, Steffen F. Siemoneit, Thomas R. Pfeiffer, Christian Gaebler, Hannes Platzgummer

**Affiliations:** * Department of Orthopaedic Surgery, Trauma Surgery, and Sports Medicine, Cologne Merheim Medical Center, Witten/Herdecke University, Cologne, Germany; † Department of Sports Orthopaedics, Klinikum Rechts der Isar, Technical University of Munich, Munich, Germany; ‡ Department of Orthopaedics, Emory University School of Medicine, Atlanta, Georgia, USA; § Sportambulatorium Wien – Zentrum fuer Orthopaedie und Sportchirurgie, Vienna, Austria; ‖ Division of Neuroradiology and Musculoskeletal Radiology, Department of Biomedical Imaging and Image-guided Therapy, Medical University of Vienna, Vienna, Austria; Investigation performed at the Division of Neuroradiology and Musculoskeletal Radiology, Department of Biomedical Imaging and Image-guided Therapy, Medical University of Vienna, Vienna, Austria

**Keywords:** knee, ACL injury, loading pattern, bone contusion, injury mechanism, prevention

## Abstract

**Background:**

Concomitant injuries after an anterior cruciate ligament (ACL) tear differ between sports, which may be related to divergent loading patterns. Bone bruises (BBs) can provide insight into the biomechanical injury mechanism.

**Purpose/Hypothesis:**

The purpose of this study was to compare BB patterns and concomitant injuries after noncontact ACL tears between pivoting sports athletes and alpine skiers. It was hypothesized that pivoting sports athletes would have a higher prevalence and depth of BBs and a higher prevalence of concomitant injuries.

**Study Design:**

Cohort study; Level of evidence, 3.

**Methods:**

A total of 446 consecutive patients with ACL injuries between December 2016 and November 2020 were retrospectively analyzed. Patients with contact injuries, an injury mechanism other than alpine skiing or pivoting sports, missing magnetic resonance imaging, failed previous nonoperative treatment, open physes, or incomplete ACL tears were excluded. Magnetic resonance imaging was used to classify BB location and depth as well as concomitant meniscal and collateral ligament injuries. There were 2 groups (alpine skiers vs pivoting sports athletes) that were propensity score matched for age, body mass index, and sex. Chi-square and Mann-Whitney *U* tests were used to detect differences, with significance set at *P* < .05. The Fleiss kappa (κ) was used to assess observer agreement.

**Results:**

Propensity score matching of 122 included patients resulted in 27 patients per group. Pivoting sports athletes showed a higher prevalence of BBs in the lateral femoral condyle than alpine skiers (85.2% vs 51.9%, respectively; *P* = .008). No significant differences were found for BB prevalence in other anatomic locations, BB depth, and concomitant meniscal and collateral ligament injuries. Post hoc power analysis showed a power of 75%. Observer agreement was almost perfect for BB prevalence (κ = 0.95-1.00), substantial for BB depth (κ = 0.68-0.75), and substantial to almost perfect for concomitant injuries (κ = 0.64-0.94).

**Conclusion:**

The prevalence of BBs in the lateral femoral condyle was higher in pivoting sports athletes than in alpine skiers after acute noncontact ACL tears. This suggests that ACL injuries in pivoting sports are associated with higher lateral compression forces in a pivot-shift mechanism, whereas anterior tibial translation and tibial rotation may be the predominant loading pattern in alpine skiing.

The main loading pattern of noncontact anterior cruciate ligament (ACL) injuries is well understood and is considered to be a multiplanar mechanism with a combination of axial compression, anterior tibial translation, valgus, and internal tibial rotation.^39,40^ This loading pattern has been described in pivoting sports and alpine skiing.^4,23^ However, recent studies have shown that concomitant injuries after ACL tears differ between alpine skiers and pivoting sports athletes. Pivoting sports athletes showed a higher prevalence of meniscal injuries, and alpine skiers had increased odds of isolated ACL injuries. It was hypothesized that different injury patterns may be related to sport-specific biomechanical injury mechanisms.^12,14^

Bone bruises (BBs) in the medial femoral condyle (MFC), lateral femoral condyle (LFC), medial tibial plateau (MTP), and lateral tibial plateau (LTP), resulting from direct tibiofemoral compression forces, can be seen on magnetic resonance imaging (MRI) in the majority of noncontact ACL injuries and have emerged as an indirect sign of the biomechanical injury mechanism.^10,36,45,50^ As a “footprint of impact,”^38,42^ BBs provide insight into the position of the knee and the energy level at the time of the ACL tear.^21,35,48^ Furthermore, previous studies have highlighted the association of BBs with concomitant intra-articular and extra-articular injuries.^2,48^ Thus, distinct BB patterns have been related to the ACL injury mechanism.^10,16,19,43^

Despite comprehensive research on ACL injuries, data on the specific patterns and implications of a BB on ACL loading patterns remain scarce. Therefore, this study aimed to investigate the differences in BB prevalence and depth as well as concomitant meniscal and collateral ligament injuries after acute noncontact ACL tears in pivoting sports athletes versus alpine skiers. It was hypothesized that (1) a higher prevalence of BBs, (2) an increased depth of BBs, and (3) a higher prevalence of concomitant injuries would occur in pivoting sports athletes compared with alpine skiers.

## Methods

### Study Population

The protocol for this study was approved by the institutional review board of the Medical University of Vienna (2354/2020). In a retrospective analysis between December 2016 and November 2020, all consecutive patients from a secondary care facility who underwent ACL surgery by a single surgeon (C.G.) were included. Patients were excluded if contact injuries, defined as physical contact with another person or object at the time of the injury, were reported.^1,25^ Patients with chronic tears, defined as failed nonoperative therapy before surgical treatment, and patients with previous ipsilateral knee joint surgery were excluded because of possible deviating BB patterns. Furthermore, partial ACL tears identified during arthroscopic surgery were excluded because of established different BB patterns.^49^ Additionally, patients were excluded if preoperative MRI was not available, the quality of MRI was insufficient (<1.5 T), the slice thickness exceeded 4 mm, or T2-weighted or proton density–weighted fat-suppressed sequences were missing in the sagittal and coronal planes. Patients were excluded if the interval between the injury and MRI exceeded 4 weeks, as volume changes in BBs occur after this period.^22,46^ Patients who suffered an ACL tear during daily activities or sports other than alpine skiing or pivoting sports were excluded. Pivoting sports included all type I and II sports according to the activity level classification,^17^ modified to European sports activities involving jumping, cutting, pivoting, or lateral movements.^33^ Finally, all patients with open growth plates were excluded during image analysis because of deviating BB patterns in skeletally immature patients.^34^

The following information was collected from all patients who met the inclusion criteria: demographic data (age, sex, and body mass index [BMI]) and injury details (type of sport, contact vs noncontact injury, date of injury, and time between injury and MRI).

### MRI Analysis

For BB analysis, all available sequences were reviewed, focusing on fat-suppressed, T2-weighted, or proton density–weighted sequences, but only true sagittal, axial, and coronal slices were considered. A BB was defined as a focal and nonlinear area of increased signal intensity on T2- or proton density–weighted fat-saturated sequences and decreased signal intensity on T1-weighted sequences, with a particular emphasis on involvement of the subchondral bone of the tibiofemoral articular surface.^31^

**Figure 1.**
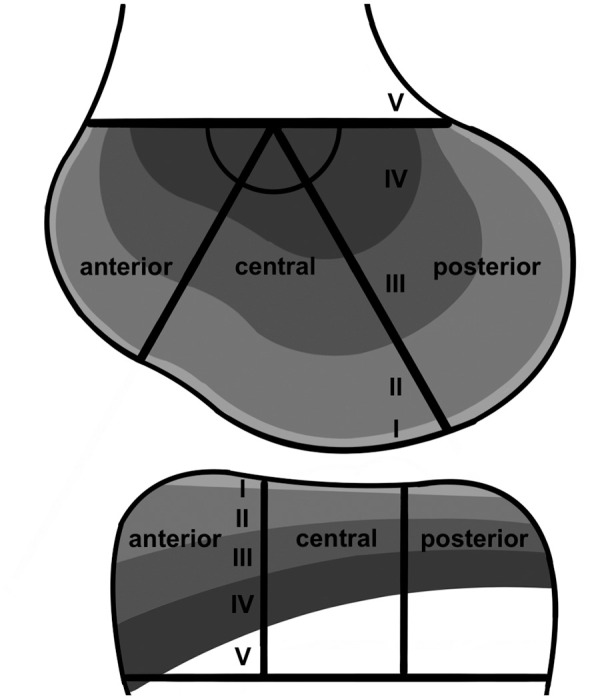
Segmentation technique according to the modified WORMS (Whole-Organ MRI Score) method.^6,37^ A horizontal line was drawn along the physeal scar. Starting from the midpoint of the femoral line, 2 lines, each at an angle of 60°, defined the borders of the anterior, central, and posterior femoral segments. At the tibia, the line was divided into anterior, central, and posterior thirds by vertical lines. Bone bruise depth was classified as superficial if it was below the subchondral bone (I), shallow if it was up to one-third of the distance to the physeal scar (II), deep if it was between one-third and two-thirds of the distance to the physeal scar (III), extensive if it was at least two-thirds of the distance to the physeal scar but did not extend beyond it (IV), or generalized if it extended beyond the physeal scar (V).^9^

The following 4 anatomic sites were assessed separately for the presence of BBs: LFC, MFC, LTP, and MTP. In addition, the aforementioned anatomic sites were divided into anterior, central, and posterior segments using the modified WORMS (Whole-Organ MRI Score) method^6,37^ (Figure 1). This method is independent of meniscus-based landmarks and was applied separately on the femur and tibia by drawing a horizontal line along the femoral and tibial physeal scars. There were 2 lines, each at a 60° angle, that were drawn from the midpoint of the femoral line to define the segment boundaries. At the tibia, the line was segmented into thirds by vertical lines. Consequently, the degree of knee flexion during MRI did not affect segmentation. The depth of the BB was categorized separately for each anatomic location according to the International Cartilage Repair Society classification system^9^ (Figure 1).

Concomitant medial collateral ligament (MCL) and lateral collateral ligament (LCL) injuries were classified into 3 grades according to Mirowitz and Shu.^32^ Only relevant structural damage (grades II and III) was included in further analysis. Medial and lateral meniscal lesions were classified according to Lotysch et al,^28^ and only grade III lesions were included.

### Measurement Protocol

A total of 3 raters, a consultant radiologist specializing in musculoskeletal radiology (H.P.), a consultant orthopaedic surgeon (C.G.), and a medical research fellow (S.T.U.), independently reviewed the MRI scans using the Horos DICOM medical image viewer (Version 4.0.0; Horos Project). All raters were blinded to demographic data, and each variable was reviewed in separate sessions. Statistical analysis was performed using the results of the consultant radiologist. There were 30 randomly selected patients compared for observer reliability analysis.

### Statistical Analysis

Data were analyzed using SPSS (Version 29.0; IBM). Propensity score matching of 1:1 was performed between the groups of alpine skiers and pivoting sports athletes. The covariates included were age, sex, and BMI. Matching was performed using the nearest neighbor approach with a caliper width of 0.2 times the standard deviation of the logit of the propensity score among eligible patients to minimize bias from confounders.^3^

Descriptive statistics including means and standard deviations were reported for each group. All continuous variables were subject to the Shapiro-Wilk test to assess distribution. Differences between groups were evaluated using the unpaired *t* test for normally distributed metric data, the Mann-Whitney *U* test for nonnormally distributed metric data, and the Pearson chi-square test for nominal data. For greater clinical relevance, BB depth was dichotomized (≤extensive vs generalized) when analyzing between-group differences. Post hoc power analysis was performed using G*Power (Version 3.1.9.7). Observer agreement was assessed using the Fleiss kappa (κ) for nominal and ordinal variables and was defined as almost perfect (κ > 0.80), substantial (κ = 0.61-0.80), moderate (κ = 0.41-0.60), fair (κ = 0.21-0.40), or poor (κ < 0.21).^27^ Significance was set at *P* < .05.

## Results

### Patient Characteristics

**Figure 2.**
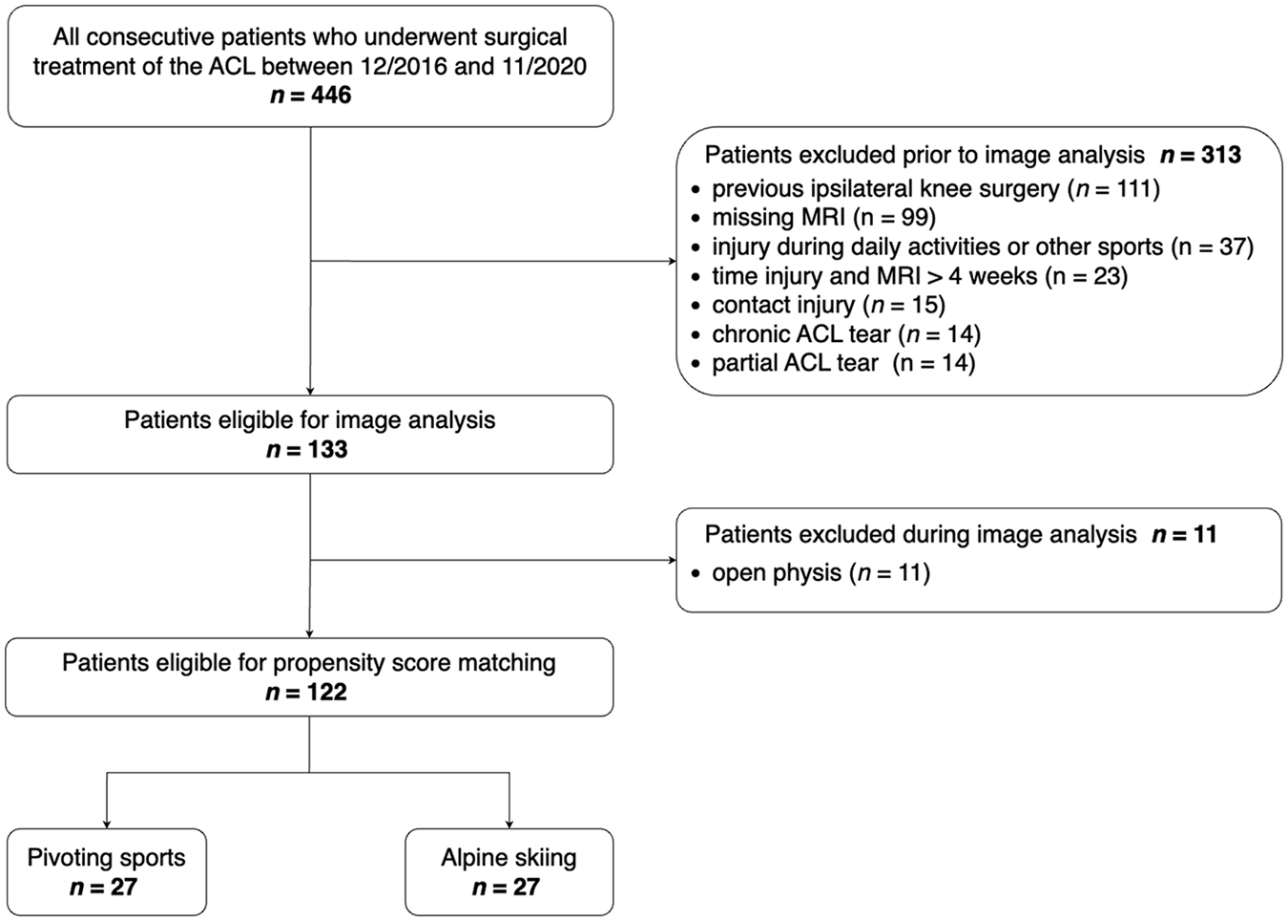
Flowchart for selecting patients. ACL, anterior cruciate ligament; MRI, magnetic resonance imaging.

After applying the exclusion criteria, a total of 122 patients were available for matching. Propensity score matching resulted in 27 alpine skiers and 27 pivoting sports athletes (Figure 2). No significant differences in terms of age, BMI, sex, and time between injury and MRI were found between the 2 groups (*P* > .05). The demographic data of the patients are presented in Table 1.

**Table 1:** Patient Characteristics^*a*^

	Alpine Skiing (n = 27)	Pivoting Sports (n = 27)	*P* Value
Age, y	31.7 ± 12.4	31.5 ± 12.1	NS
BMI, kg/m^2^	23.3 ± 2.9	24.0 ± 3.0	NS
Sex			NS
Female	14 (51.9)	12 (44.4)	
Male	13 (48.1)	15 (55.6)	
Time from injury to MRI, d	3.5 ± 2.6	4.6 ± 5.6	NS
Type of sport			—
Skiing	27 (100.0)	—	
Soccer	—	10 (37.0)	
Racket sports	—	6 (22.2)	
Handball	—	3 (11.1)	
Basketball	—	3 (11.1)	
Gymnastics	—	2 (7.4)	
Dancing	—	2 (7.4)	
Football	—	1 (3.7)	

a Data are presented as mean ± SD or n (%). BMI, body mass index; MRI, magnetic resonance imaging; NS, not significant.

### MRI Analysis

Overall, BBs were found in 92.6% of patients. No significant difference was found in the overall presence of BBs between the alpine skiers and pivoting sports athletes (96.3% vs 88.9%, respectively; *P* > .05). The most frequently affected anatomic site was the LTP (90.7%), followed by the LFC (68.5%), the MFC (55.6%), and the MTP (50.0%). While the most affected anatomic site in alpine skiers was the LTP (96.3%), followed by the MFC (63.0%), pivoting sports athletes showed the highest BB prevalence in the LTP and LFC (both 85.2%). The prevalence of BBs in the LFC was significantly higher in pivoting sports athletes than in alpine skiers (85.2% vs 51.9%, respectively; *P* = .008). An in-depth analysis of the BB distribution regarding the segments showed a tendency toward a more anterior aspect of the MTP for pivoting sports athletes (Table 2). No significant differences were found between the groups regarding the BB presence in other anatomic sites (*P* > .05). In addition, there were no significant differences (*P* > .05) between the groups in terms of the BB depth (Table 3).

**Table 2:** BBs and Concomitant Injuries^*a*^

	Alpine Skiing (n = 27)	Pivoting Sports (n = 27)	*P* Value
BB in MFC	17 (63.0)	13 (48.2)	NS
Anterior	00 (0.0)	00 (0.0)	
Central	14 (51.9)	11 (40.7)	
Posterior	3 (11.1)	2 (7.4)	
BB in LFC	14 (51.9)	23 (85.2)	.008
Anterior	00 (0.0)	00 (0.0)	
Central	14 (51.9)	22 (81.5)	
Posterior	00 (0.0)	2 (7.4)	
BB in MTP	12 (44.4)	15 (55.6)	NS
Anterior	00 (0.0)	2 (7.4)	
Central	00 (0.0)	1 (3.7)	
Posterior	12 (44.4)	13 (48.2)	
BB in LTP	26 (96.3)	23 (85.2)	NS
Anterior	1 (3.7)	1 (3.7)	
Central	00 (0.0)	00 (0.0)	
Posterior	26 (96.3)	23 (85.2)	
Medial meniscal injury	8 (29.6)	9 (33.3)	NS
Lateral meniscal injury	2 (7.4)	2 (7.4)	NS
MCL injury	6 (22.2)	4 (14.8)	NS
LCL injury	1 (3.7)	1 (3.7)	NS

a Data are presented as n (%). BB, bone bruise; LCL, lateral collateral ligament; LFC, lateral femoral condyle; LTP, lateral tibial plateau; MCL, medial collateral ligament; MFC, medial femoral condyle; MTP, medial tibial plateau; NS, not significant.

**Table 3:** Bone Bruise Depth^*a*^

	Alpine Skiing	Pivoting Sports	*P* Value
MFC			NS
≤Extensive	16 (94.1)	13 (100.0)	
Generalized	1 (5.9)	00 (0.0)	
LFC			NS
≤Extensive	10 (71.4)	19 (82.6)	
Generalized	4 (28.6)	4 (17.4)	
MTP			NS
≤Extensive	6 (50.0)	10 (66.7)	
Generalized	6 (50.0)	5 (33.3)	
LTP			NS
≤Extensive	4 (15.4)	9 (39.1)	
Generalized	22 (84.6)	14 (60.9)	

a Data are presented as n (%). The chi-square test was used with dichotomized values for bone bruise depth (≤extensive vs generalized). LFC, lateral femoral condyle; LTP, lateral tibial plateau; MFC, medial femoral condyle; MTP, medial tibial plateau; NS, not significant.

Overall, concomitant injuries occurred more frequently on the medial side (medial meniscus: 31.5% [n = 17]; MCL: 18.5% [n = 10]) than on the lateral side (lateral meniscus: 7.4% [n = 4]; LCL: 3.7% [n = 2]). There was no significant difference in the distribution of concomitant injuries associated with ACL tears when comparing alpine skiing to pivoting sports (*P* > .05) (Table 2). With an effect size of 0.359, post hoc power analysis showed a power of 75%.

### Observer Agreement

Interrater agreement on the presence of BBs at each anatomic site was almost perfect (κ = 0.95-1.00), and agreement on BB depth was substantial (κ = 0.68-0.75). Regarding concomitant injuries, agreement was substantial for the LCL (κ = 0.64) and lateral meniscus (κ = 0.71) and almost perfect for the MCL (κ = 0.83) and medial meniscus (κ = 0.94).

## Discussion

The main finding of this study was that the prevalence of BBs in the LFC was significantly higher in pivoting sports athletes than in alpine skiers after acute noncontact ACL tears. However, we did not observe differences in BB depth and concomitant injuries between the groups.

The current literature suggests that the main loading pattern on the knee in noncontact ACL injuries is a multiplanar loading mechanism with a combination of axial compression, anterior tibial translation, valgus, and internal tibial rotation,^39,40^ which has been described in systematic video analyses in pivoting sports and alpine skiing.^4,23^ However, recent studies have shown that concomitant injuries after ACL tears vary between alpine skiers and soccer players. A retrospective analysis of surgical reports and arthroscopic images of elite professional alpine skiers and soccer players undergoing primary ACL reconstruction showed that pivoting sports athletes demonstrated a higher prevalence of medial meniscal tears and lateral posterior meniscus root lesions.^12^ Furthermore, alpine skiers showed increased odds of isolated ACL injuries compared with pivoting sports athletes.^14^ Both studies hypothesized that different injury patterns may be related to sport-specific biomechanical injury mechanisms.^12,14^ For example, the distinct mechanisms described in alpine skiing—the slip-catch, dynamic snowplow, and landing back-weighted—and the ski acting as a lever may result in different knee loading patterns compared with the rapid directional changes in high shoe-surface interaction seen in pivoting sports.^4^

**Figure 3.**
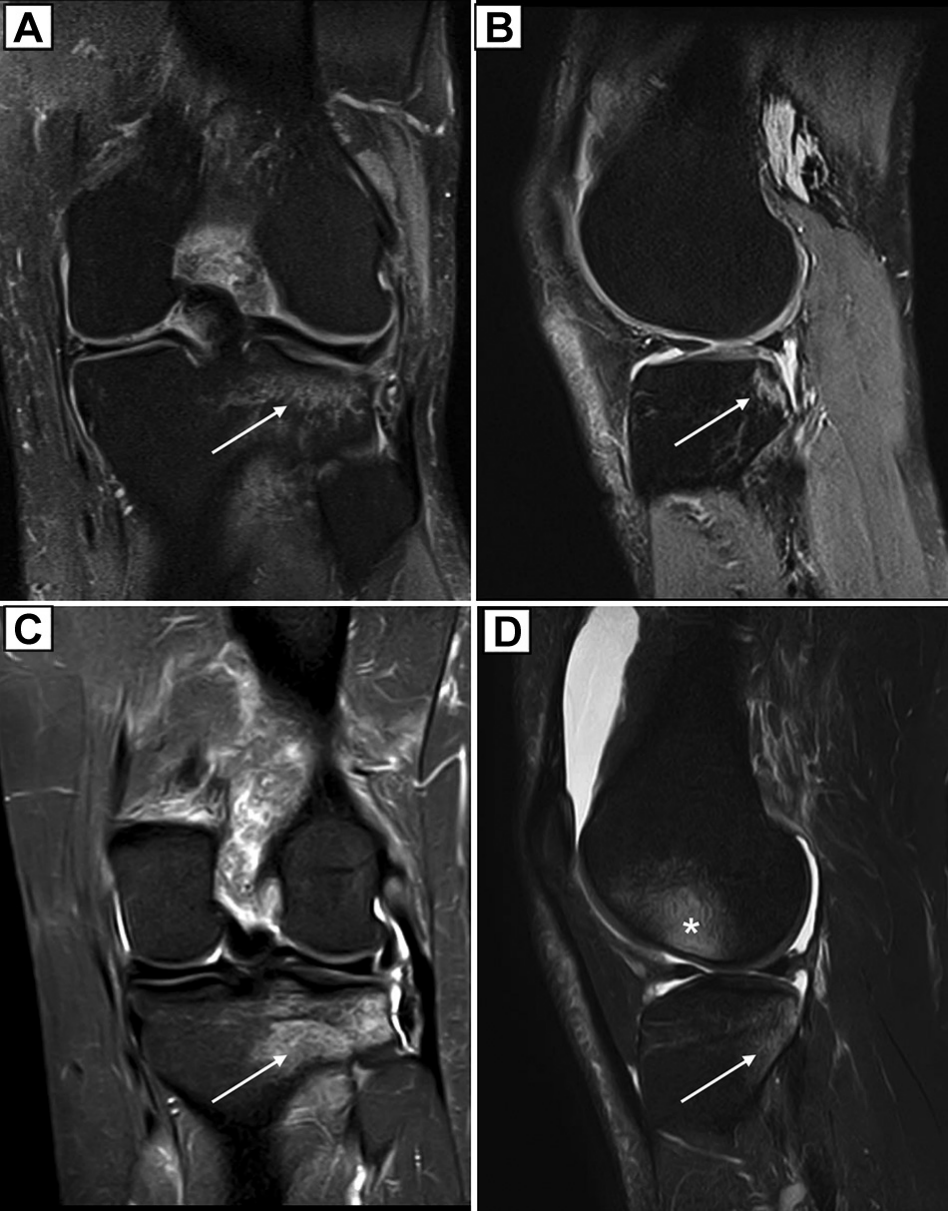
Proton density–weighted fat-saturated magnetic resonance imaging of the left knee in an alpine skier (A: coronal; B: sagittal) and a soccer player (C: coronal; D: sagittal), highlighting the predominant distribution of bone bruises (BBs) after anterior cruciate ligament (ACL) tears. The images show BBs in the lateral tibial plateau (arrow) and a higher prevalence of BBs in the lateral femoral condyle (asterisk) in athletes engaged in pivoting sports.

The results of this study support this hypothesis. Pivoting sports athletes showed a significantly higher prevalence of BBs in the LFC than alpine skiers (Figure 3). This lateral BB pattern in pivoting sports is consistent with previous studies in pivoting sports athletes^10,44^ and is associated with increased knee valgus loading during an injury.^42,50^ Furthermore, involvement of the LFC is associated with an increased risk of MCL lesions,^24^ which is the primary valgus stabilizer.^15^ This suggests a lower knee valgus angle at the time of the injury in alpine skiing compared with pivoting sports, which is consistent with video analyses showing less pronounced knee valgus changes compared with knee flexion and internal rotation of the tibia in alpine skiers.^5^ Furthermore, BB analysis revealed that pivoting sports athletes showed BBs in the central and anterior aspects of the MTP. Considering that the origin of medial BBs has not been definitively explained, our study findings support the hypothesis that medial BBs are the result of a contrecoup injury after a pivot-shift injury. The high compression force is not dissipated by the initial pivot-shift mechanism and results in a compensatory varus alignment with internal rotation of the femur, with the MTP remaining subluxation anteriorly.^19^

The results of BB depth analysis are somewhat inconsistent with the aforementioned findings, as BB depth is thought to be related to the energy level of the injury, that is, the compression force.^7,47,48^ However, this study used a semiquantitative measurement method to account for the potential variability in MRI sequences from different institutions, and differences may not have been detected with this measurement method. Future studies should focus on quantitative measures to analyze differences in BB severity.

In summary, the results of BB analysis and their agreement with the previously mentioned findings from the study by Farinelli et al^12^ suggest that in alpine skiing, anterior shearing and tibial torque may be the predominant loading pattern in ACL injuries, whereas in pivoting sports, a pivot-shift mechanism with excessive lateral compression and valgus force is present.

In terms of concomitant injuries, this study found no significant differences between the 2 groups. This contrasts with previous studies in which soccer players had a higher rate of medial meniscal tears and lateral posterior meniscus root lesions compared with skiers. The reasons for this may be as follows: First, this is an MRI-based study. It is well known that the sensitivity of MRI for meniscal lesions, especially the medial and lateral meniscus roots, is limited.^20,26^ Second, the patients in this study were not only elite athletes, in whom the mechanisms of injury of ACL tears might be different from those seen in recreational athletes.^12^ Furthermore, the reported prevalence of concomitant injuries in patients with ACL tears varies widely between studies.^12,14,18,44^ With these points in mind, the predictive value of concomitant meniscal and collateral ligament injuries on the injury mechanism in this study is restricted.

The clinical value of this study is that it supports recent recommendations for sport-specific injury prevention programs because of the different biomechanical injury mechanisms between pivoting sports and alpine skiing based on BB analysis.^13,29,30^ The effectiveness of ACL injury prevention programs has recently been demonstrated in 2 meta-analyses.^11,41^ However, the focus of the studies reviewed was on pivoting sports, particularly soccer. This has highlighted the importance of more sport-specific data in ACL prevention strategies.^11^ Therefore, the Ligament Committee of the German Knee Society recommends that prevention programs be adapted to different sports disciplines.^30^ In addition, programs should target high-risk movements within a specific sport rather than implementing a one-size-fits-all program.^29^ The results of this study are valuable in the development and improvement of sport-specific injury prevention programs with an awareness of sport-specific injury patterns. It has been hypothesized that pivoting sports athletes may benefit from a focus on running and plyometric exercises, whereas alpine skiers may benefit from strength training.^8,30^ However, it is important that prevention programs target multiplanar movements that address the complex loading pattern of the ACL rather than single-plane mechanics. In addition, internal and external risk factors should be considered, and athlete motivation and compliance should be emphasized.^29,30,40^

### Limitations

There are some limitations to this study. First, as the patients were recruited from a secondary care facility and because of the retrospective nature of the study, selection bias cannot be ruled out. This is the reason for the lack of a higher incidence of more severe multiligamentous injuries. Second, the group of pivoting sports included 7 different sports disciplines that were classified as type I or II sports according to the activity level classification modified to European sports activities.^33^ This heterogeneity within the group may affect optimal propensity score matching by introducing confounding factors in BB location. However, a previous study found no significant differences in BB location or meniscal injuries after ACL tears between different pivoting sports, suggesting a similar biomechanical loading pattern.^44^ Third, the analysis of BB depth may have been affected by the inclusion of MRI scans from different institutions. A semiquantitative technique was used to measure BB depth to account for the potential variability of MRI. Finally, BBs can only present a static depiction of the injury mechanism, with limited information on accompanying risk factors or other joint positions at the time of the injury. The limitations of different research methodologies in providing insight into the underlying injury mechanisms have been outlined previously.^25^ Future studies should focus on correlating BB location with an analysis of the ACL injury based on video recordings. In addition, a larger study population would allow a more detailed analysis of the differences between pivoting sports athletes and alpine skiers regarding the anteroposterior location of the BB.

## Conclusion

The prevalence of BBs in the LFC was higher in pivoting sports athletes than in alpine skiers after acute noncontact ACL tears. This suggests that ACL injuries in pivoting sports are associated with higher lateral compression forces in a pivot-shift mechanism, whereas in alpine skiing, anterior tibial translation and tibial rotation may be the predominant loading pattern. These findings may be beneficial for the improvement of sport-specific injury prevention programs.
